# Aberrant CD200/CD200R1 expression and function in systemic lupus erythematosus contributes to abnormal T-cell responsiveness and dendritic cell activity

**DOI:** 10.1186/ar3853

**Published:** 2012-05-23

**Authors:** Yang Li, Li-dan Zhao, Lu-sha Tong, Su-ning Qian, Yan Ren, Lei Zhang, Xin Ding, Yang Chen, Yan-xia Wang, Wen Zhang, Xiao-feng Zeng, Feng-chun Zhang, Fu-lin Tang, Xuan Zhang, De-nian Ba, Wei He, Xue-tao Cao, Peter E Lipsky

**Affiliations:** 1 Department of Rheumatology & Clinical Immunology, Peking Union Medical College Hospital, Chinese Academy of Medical Sciences and Peking Union Medical College, 41# Da-Mu-Cang-Hu-Tong Street, Beijing 100032, China; 2 Department of Immunology, School of Basic Medicine, Peking Union Medical College, Institute of Basic Medical Sciences, Chinese Academy of Medical Sciences, #5 Dong-Dan-San-Tiao, Beijing 100005, China; 3 Formerly National Institute of Arthritis and Musculoskeletal and Skin Diseases, National Institutes of Health, 9000 Rockville Pike, Bethesda, MD 20892, USA

## Abstract

**Introduction:**

CD200 is a type I transmembrane glycoprotein that can regulate the activation threshold of inflammatory immune responses, polarize cytokine production, and maintain immune homeostasis. We therefore evaluated the functional status of CD200/CD200 receptor 1 (CD200R1) interactions in subjects with systemic lupus erythematosus (SLE).

**Methods:**

Serum CD200 level was detected by ELISA. The expression of CD200/CD200R1 by CD4^+ ^T cells and dendritic cells (DCs) was examined by flow cytometry, and then compared between SLE patients and healthy controls. Peripheral blood mononuclear cells were stained with carboxyfluorescein diacetate succinimidyl ester and annexin V/propidium iodide for evaluation of the effect of CD200 on cell proliferation and apoptosis. In addition, the effect of CD200 on DC function was determined by transwell migration assay as well as by measurement of binding and phagocytosis of apoptotic cells.

**Results:**

In SLE patients, the number of CD200^+ ^cells and the level of soluble CD200 were significantly higher than in healthy controls, whereas the expression of CD200R1 by CD4^+ ^T cells and DCs was decreased. Furthermore, the increased CD200 expression by early apoptotic cells contributed to their diminished binding and phagocytosis by DCs in SLE. Importantly, the engagement of CD200 receptor on CD4^+ ^T cells with CD200-Fc fusion protein *in vitro *reduced the differentiation of T-helper type 17 cells and reversed the defective induction of CD4^+^CD25^high^FoxP3^+ ^T cells by transforming growth factor beta in SLE patients. Conversely, blockade of CD200-CD200R1 interaction with anti-CD200R1 antibody promoted CD4^+ ^T-cell proliferation.

**Conclusion:**

CD200 and CD200R1 expression and function are abnormal in SLE and may contribute to the immunologic abnormalities in SLE.

## Introduction

Systemic lupus erythematosus (SLE) is an autoimmune disease that affects many tissues and organs. The major immunopathological findings of SLE include defective immune regulation with the breakdown of immune tolerance, autoantibody formation followed by immune complex deposition, cytokine imbalance, and inflammation [1]. Failure of phagocytes to remove apoptotic cells has been suggested to allow excessive release of autoantigens and to lead to the induction of autoimmunity, although the underlying mechanisms remain unclear [2-6]. In addition, CD4^+^CD25^high^FoxP3^+ ^regulatory T cells (Tregs), which are pivotal in the maintenance of T-cell homeostasis and are critical regulators of immune tolerance [7,8], exhibit quantitative and/or qualitative deficiencies in SLE that may contribute to the development of lupus pathogenesis [9,10].

CD200 is a type I transmembrane glycoprotein belonging to the immunoglobulin superfamily, and is expressed by a variety of cells, including B cells, activated T cells, follicular dendritic cells (DCs), and neurons [11-13]. CD200 consists of extracellular, transmembrane, and intracellular domains, although its intracellular region lacks a signaling motif [11-13]. CD200 receptors include CD200R1 to CD200R4, of which CD200R1 has the highest binding affinity [14]. The distribution of CD200 receptors is mainly on myeloid-derived cells, such as DCs, macrophages, and also activated T cells [15,16]. The known immunoregulatory roles of the CD200/CD200R1 pathway include suppression of the degranulation of mast cells and basophils [17] and negative regulation of macrophage function [18].

Hoek and colleagues found that CD200-deficient mice had increased endogenous activation of macrophages/myeloid cells in the central nervous system, with enhanced susceptibility to experimental allergic encephalomyelitis and collagen-induced arthritis [18]. Administration of CD200R-Ig to disrupt CD200-CD200R interaction also increased the susceptibility of mice to collagen-induced arthritis. Furthermore, Broderick and colleagues reported that blockade of CD200 resulted in the early onset of experimental autoimmune uveoretinitis in mice [19]. In addition, Rosenblum and colleagues studied CD200-knockout mice in a model of UV-mediated induction of tolerance to hapten, and suggested that the expression of CD200 in skin cells plays a role in autoimmune congenital alopecia [20]. Finally, Gorczynski and colleagues showed that tumor growth *in vivo *can be monitored by levels of soluble CD200 in serum of tumor-bearing animals [21], whereas Moreaux and colleagues found significant overexpression of CD200 in a variety of cancers compared with normal cells or tissues and suggested that CD200 might be a potential therapeutic target and prognostic factor for a large array of malignancies [22].

While available evidence highlighted an important role of CD200/CD200R1 in experimental autoimmune diseases, the role of CD200/CD200R1 in human autoimmune diseases such as SLE remains unknown. We therefore explored the expression and function of CD200/CD200R1 in subjects with SLE.

## Materials and methods

### Patients and healthy controls

Altogether, a total of 161 new-onset untreated patients fulfilling the American College of Rheumatology classification criteria for SLE were enrolled in this study. All were female, and their age ranged from 12 to 55 years with a mean age of 29.0 ± 10.2 years (see Additional file 1). Ninety-five gender-matched and age-matched healthy volunteers were recruited as healthy controls (HCs). The Ethics Committee of Peking Union Medical College Hospital approved this study and informed consent was obtained from each patient and HC.

### Antibodies and reagents

mAbs targeting the following molecules were used, either unlabeled or as fluorescein isothiocyanate, phycoerythrin, allophycocyanin, or phycoerythrin-cyanin 7 conjugates: CD4 (RPA-T4), CD11c (3.9), CD25 (BC96), CD200R (OX-108), CD38 (HIT2), and CD123 (6H6) from BioLegend (San Diego, CA, USA); IFNγ (B27), CD3 (UCHT1), and CD28 (CD28.2) from BD Pharmingen (Franklin Lakes, NJ, USA); and CD200 (OX104), IL-4 (MP4-25), IL-17 (eBio64DEC17), and Foxp3 (PCH101) from eBioscience (San Diego, CA, USA). In all experiments, control mAbs of the respective IgG were included.

The CD200 Duoset and B-cell activating factor belonging to the TNF family, IFNα, and IL-6 ELISA kits were purchased from R&D Systems (Minneapolis, MN, USA). The Cell Trace™ carboxyfluorescein diacetate succinimidyl ester cell proliferation kit was obtained from Invitrogen (Carlsbad, CA, USA). Recombinant IL-2, IL-4, transforming growth factor beta (TGF-β) 1, and granulocyte-macrophage colony-stimulating factor (GM-CSF) were purchased from PeproTech Inc. (Rocky Hill, NJ, USA); PKH26, PKH67, ionomycin, and phorbol-12-myristate-13-acetate were obtained from Sigma-Aldrich (St Louis, MO, USA); the Vybrant Apoptosis Assay Kit #2 was obtained from Invitrogen; and recombinant human CD200-Fc, anti-human CD200 R1 antibody, and human IgG control were purchased from R&D Systems.

### Enzyme-linked immunosorbent assay

The levels of serum CD200, IL-6, IFNα, and B-cell activating factor belonging to the TNF family were detected with ELISA kits according to the manufacturer's instructions.

### Real-time polymerase chain reaction

Total RNA was isolated from peripheral blood mononuclear cells (PBMC) of SLE patients and HCs with TRIzol (Invitrogen), first-strand cDNA was synthesized, and the DNA amplification was detected with the dye SYBR Green (TAKARA, Otsu, Japan). Primers were as follows: for CD200, 5'-CCGTCAACAAAGGCTATTGG-3' (forward) and 5'-ATTTAGGGCTCTCGGTCCTG-3' (reverse); for CD200R1, 5'-GACCAGAGAGGGTCTCACCA-3' (forward) and 5'-TTGAAGCGGCCACTAAGAAG-3' (reverse); and for glyceraldehyde-3-phosphate dehydrogenase, 5'-ACTTCAACAGCGACACCCACT-3' (forward) and 5'-GCCAAATTCGTTGTCATACCAG-3' (reverse).

### Cell culture, stimulation and treatment

Cells were cultured in RPMI 1640 medium supplemented with 100 U/ml penicillin and 100 μg/ml streptomycin, 0.05 mM nonessential amino acids, 2 mM L-glutamine, as well as 10% heat-inactivated FCS in a humidified carbon dioxide-containing atmosphere at 37°C. Cells were stimulated with anti-CD3 and anti-CD28 mAbs at 1 μg/ml, respectively. Recombinant human CD200-Fc, anti-human CD200R1 antibody, and human IgG control were used at 100 ng/ml; and recombinant human IL-2, TGF-β, IL-4, and GM-CSF were used at 20 ng/ml, 2 ng/ml, 100 ng/ml, and 100 ng/ml, respectively. For T-cell differentiation experiments, PBMC were co-cultured with CD200-Fc or anti-CD200R1 for 48 hours. Golgistop was added in the presence of phorbol-12-myristate-13-acetate and ionomycin 5 hours before cells were collected and stained for membrane molecules. Intracellular staining for IL-17, IL-4, and IFNγ was also performed after fixation and permeabilization with fixation/permeabilization buffer. For cell proliferation assays, PBMC were stained with 5 μM carboxyfluorescein diacetate succinimidyl ester, stimulated by anti-CD3 and anti-CD28 mAbs alone or in the presence of CD200-Fc or anti-CD200R1, and cell proliferation was measured on day 5 by flow cytometry. The cell division index - which was defined as the ratio of the proportion of proliferated cells with decreased carboxyfluorescein diacetate succinimidyl ester fluorescence after stimulation to that without stimulation - was analyzed.

### Cell separation

CD4^+^CD25^- ^T cells and CD14^+ ^monocytes were isolated using a CD4^+^CD25^+ ^Regulatory T Cell Isolation Kit (130-091-301; Miltenyi Biotec) and CD14 MicroBeads (130-050-201; Miltenyi Biotec) according to the manufacturer's instructions. PBMC were induced to undergo apoptosis by X-ray accelerator (5 Gray) [23] and cultured in complete RPMI 1640 medium in a humidified carbon dioxide-containing atmosphere at 37°C for 48 hours. Apoptosis and necrosis were detected by annexin V and propidium iodide (PI) staining according to the manufacturer's protocol. CD200^+ ^live cells, CD200^- ^live cells, CD200^+ ^apoptotic cells, CD200^- ^apoptotic cells, and necrotic cells were sorted by flow cytometry using the Moflo (Cytomation, Fort Collins, Colorado, USA). The sort gates were additionally restricted to the lymphocyte gate as determined by typical forward and sideward scatter characteristics [24,25].

### Generation of dendritic cells

Monocytes were cultured with recombinant human GM-CSF (100 ng/ml) and recombinant human IL-4 (100 ng/ml) and were harvested after 6 days. The monocyte-derived DCs were used for co-culture experiments and transwell assays.

### Cell staining and co-culture

The monocyte-derived DCs and apoptotic and necrotic cell targets were labeled with green fluorescent dye PKH67 and red fluorescent dye PKH26 (Sigma-Aldrich), respectively, and then co-cultured (cell ratio 10^4^:10^4^) for 3 hours, after which they were analyzed by fluorescence microscopy and flow cytometry.

### Transwell migration assay

The monocyte-derived DCs were seeded in the upper chambers of the transwell. The lower chambers were filled with IgG control (100 ng/ml), CD200Fc (100 ng/ml), RANTES (50 ng/ml) or CD200Fc plus RANTES. After 8 hours of incubation, the cells that had migrated to the lower chamber were counted.

### Western blot

CD4^+ ^T cells were cultured with recombinant human CD200Fc (IgG as control). After 5 days, cells were harvested, washed twice in ice-cold PBS, and lysed by incubation for 1 hour in a buffer containing 20 mM HEPES (pH 7.9), 20% glycerol, 1% Nonidet P-40, 1 mM MgCl_2_, 0.5 mM ethylenediamine tetraacetic acid, 0.1 mM ethyleneglycol tetraacetic acid, 1 mM dithiothreitol, 1 mM phenylmethylsulfonyl fluoride and a proteinase inhibitor cocktail (BD Biosciences, Franklin Lakes, NJ, USA). Lysates were kept on ice and vortexed every 10 minutes for 1 hour before centrifugation at 13,000 rpm and 4°C. Equal amounts of protein were separated by SDS-PAGE (Invitrogen) and transferred to Immobilon PVDF membranes (Millipore, Billerica, Massachusetts, USA) and were blocked with 5% dry milk in PBS containing 0.5% Tween 20 before incubation with specific antibodies against downstream of tyrosine kinase 2 (DOK2; ab47507) or phosphorylated DOK2 (phospho Y299) (ab47376; Abcam, Cambridge, UK), followed by incubation with horseradish peroxidase-conjugated secondary antibody and development using Western Lightning Chemiluminescence Reagent Plus (PerkinElmer Life Sciences, Inc. Waltham, Massachusetts, USA).

### Statistical analysis

All data were analyzed using SPSS 13.0 software. Data that passed both the Kolmogorov-Smirnov and the Shapira-Wilk tests (*P *> 0.05) were considered in a normal distribution. For data with normal distribution and homogeneity of variance (means and standard deviations), one-way analysis of variance with adjusted Bonferroni correction was used to assess the differences among groups. An independent-sample *t *test and a paired-sample *t *test were used to compare differences between two groups and differences before and after treatment. Correlation was calculated with Pearson's correlation. For data with a non-normal distribution (medians and 25th to 75th percentiles), the Mann-Whitney test was used to compare differences between two groups and correlation was analyzed with Spearman's rank-order test. *P *< 0.05 was considered statistically significant.

## Results

### Increased CD200 but decreased CD200R1 expression by CD4^+ ^T cells and dendritic cells in SLE patients

**Figure 1:**
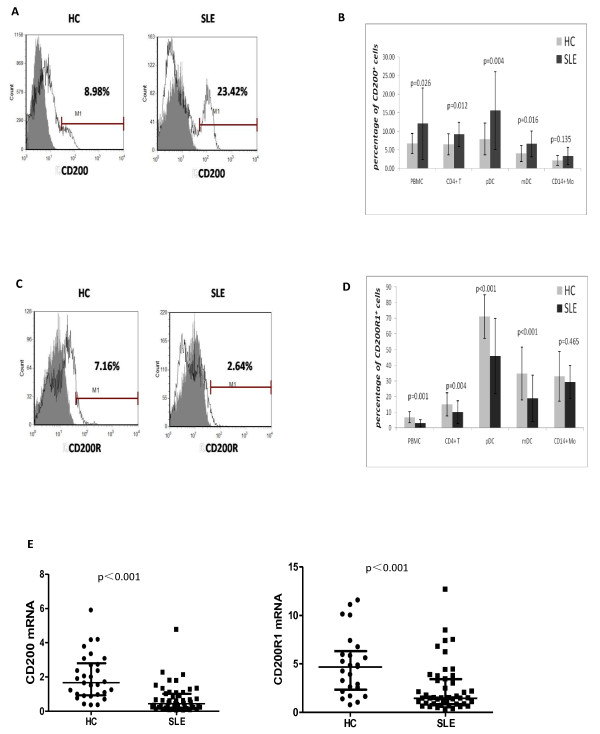
**CD200 and CD200 receptor 1 expression in systemic lupus erythematosus patients and healthy controls**. **(A) **CD200 expression in peripheral blood mononuclear cells (PBMC) was upregulated in systemic lupus erythematosus (SLE) patients. Open histogram: percentage of CD200^+ ^cells in PBMC; gray histogram, IgG control. **(B) **Percentage of CD200^+ ^cells in PBMC (12.03 ± 9.67% vs. 6.68 ± 2.69%, *P *= 0.026), CD4^+ ^T cells (9.17 ± 3.26% vs. 6.45 ± 2.82%, *P *= 0.012), CD14^+ ^monocytes, CD11c^-^CD123^high ^plasmacytoid dendritic cells (pDCs) (15.57 ± 10.48% vs. 7.89 ± 4.26%, *P *= 0.004) and CD11c^+^CD123^- ^myeloid dendritic cells (mDCs) (6.55 ± 3.46% vs. 4.01 ± 2.10%, *P *= 0.016) from SLE patients (*n *= 53) and healthy controls (HCs) (*n *= 24). **(C) **CD200 receptor 1 (CD200R1) expression in PBMC was downregulated in SLE patients. Open histogram: percentage of CD200R1^+ ^cells in PBMC; gray histogram, IgG control. **(D) **Percentage of CD200R1^+ ^cells in PBMC (3.10 ± 2.24% vs.6.88 ± 3.61%, *P *= 0.001), CD4^+ ^T cells (10.11 ± 7.37% vs. 15.08 ± 7.50%, *P *= 0.004), CD14^+ ^monocytes, CD11c^-^CD123^high ^pDCs (45.93 ± 24.07% vs. 71.28 ± 13.91%, *P *< 0.001) and CD11c^+^CD123^- ^mDCs (18.91 ± 14.90% vs. 34.75 ± 16.82%, *P *< 0.001) from SLE patients (*n *= 53) and HCs (*n *= 24). **(E) **mRNA expression of both CD200 (median 0.44, interquartile range 0.20 to 1.02 vs. 1.67, 0.93 to 2.80; *P *< 0.001) and CD200R1 (median 1.45, interquartile range 0.85 to 3.42 vs. 4.69, 2.33 to 6.33; *P *< 0.001) in PBMC was significantly lower in SLE patients than in HCs.

**Figure 2:**
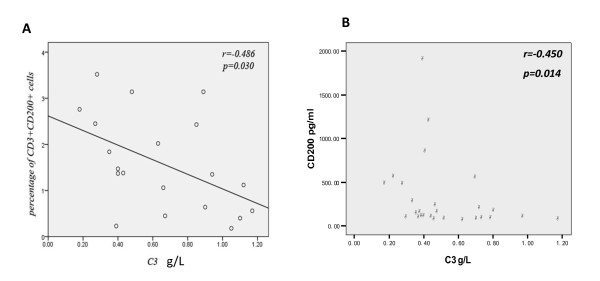
**Correlation between CD200 and complement 3**. **(A) **In systemic lupus erythematosus (SLE) patients, the proportion of CD3^+^CD200^+ ^cells was negatively correlated with the serum complement 3 (C3) level (*r *= -0.486, *P *< 0.05). **(B) **Serum CD200 level was negatively correlated with serum C3 in SLE patients (*r *= -0.45, *P *= 0.014).

CD200 and CD200R1 expression was analyzed in SLE patients and HCs. The proportion of CD200^+ ^cells in PBMC of SLE patients was significantly higher than that in HCs (Figure 1A, B), especially in the CD4^+ ^T-cell population, the CD11c^-^CD123^high ^plasmacytoid DCs, and the CD11c^+^CD123^- ^myeloid DCs (Figure 1B; see Additional file 2), but not in CD8^+ ^T cells, CD19^+ ^B cells, CD38^bright ^plasma cells (data not shown), or CD14^+ ^monocytes (*P *> 0.05). The percentage of CD3^+^CD200^+ ^cells was negatively correlated with serum complement 3 (*r *= -0.486, *P *< 0.05; Figure 2A). Unlike the cell numbers, there was no significant difference in the mean fluorescence intensities of CD200 expression between SLE patients and HCs in the different cell subgroups (*P *> 0.05). Finally, even though the frequency of CD200-expressing cells was increased in SLE, CD200 mRNA expression in PBMC was significantly lower in SLE patients than in HCs (Figure 1E).

In keeping with the increased percentage of cells expressing CD200, the circulating levels of CD200 in SLE patients were also significantly higher than that in HCs (median 142.22, interquartile range 82.26 to 469.29 vs. 76.62, 45.9 to 124.93 pg/ml; *P *= 0.001). Furthermore, the serum CD200 level negatively correlated with the serum complement 3 level (*r *= -0.45, *P *= 0.014; Figure 2B), but not the Systemic Lupus Erythematosus Disease Activity Index score or the levels of serum B-cell activating factor belonging to the TNF family, IL-6, IFNα, or anti-dsDNA (*P *> 0.05; see Additional file 3).

In contrast to CD200 expression, SLE patients had a decreased proportion of CD200R1^+ ^cells in PBMC compared with HCs. This reduction was evident in CD4^+ ^T cells, plasmacytoid DCs, and myeloid DCs (Figure 1C, D). CD200R1 mRNA expression in PBMC was also significantly lower in SLE patients than in HCs (Figure 1E). Of note, CD4^+^CD45RA^+ ^naïve T cells had less CD200R1 expression than CD4^+^CD45RO^+ ^memory T cells in both HCs and SLE patients (*P *< 0.05), and there was no significant difference between SLE patients and HCs (see Additional file 4).

### Anti-CD200R1 antibody promotes T-cell receptor-driven proliferation of CD4^+ ^T cells in SLE patients

Next we set out to determine whether the defective CD200R1 expression by CD4^+ ^T cells affects CD4^+ ^T-cell function in SLE patients. As engagement of CD200R1 by CD200 is known to initiate signaling by inducing phosphorylation of docking protein 2 or the adaptor protein DOK2 (p56doc-2) [26], we initially determined whether soluble CD200Fc could influence CD200/CD200R1 signaling in CD4^+ ^T cells by examining its effect on phosphorylation of DOK2. We found that CD200Fc induced phosphorylation of DOK2 in CD4^+ ^T cells. This result is consistent with previously reported findings and indicates that soluble CD200Fc by engaging CD200R is an agonist of the CD200/CD200R1 signaling pathway, whereas anti-CD200R1 antibody, according to the product instructions, is an antagonist and can block the receptor-ligand interaction (see Additional file 5).

**Figure 3:**
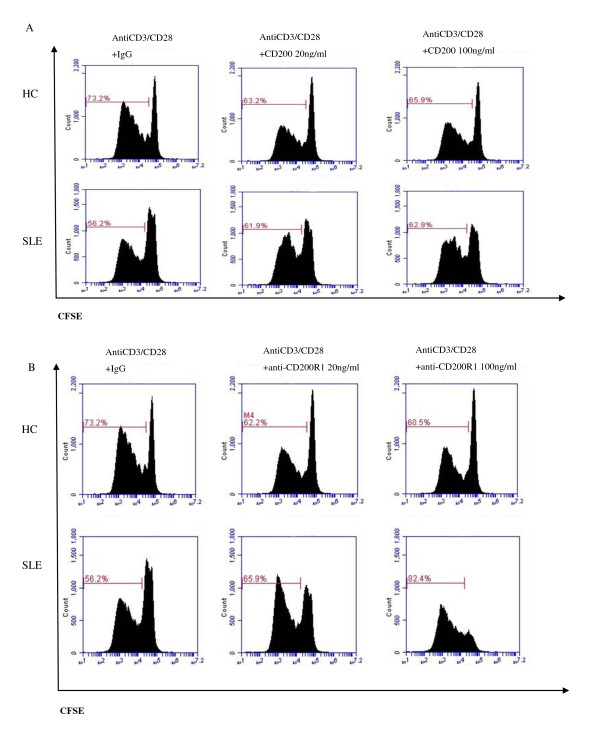
**Anti-CD200R1 antibody promoted T-cell receptor-driven proliferation of CD4^+ ^T cells in systemic lupus erythematosus patients**. **(A) **In systemic lupus erythematosus (SLE) patients and healthy controls (HCs), no significant difference in proliferation of anti-CD3/CD28-stimulated CD4^+ ^T cells was found in the presence of CD200Fc or IgG control. **(B) **In SLE patients, anti-CD200R1 promoted anti-CD3/CD28-stimulated proliferation of CD4^+ ^T cells in a concentration-dependent manner, whereas this was not observed in HCs. Fluorescence-activated cell sorting (OK)plots are representative of five experiments performed on five SLE patients and HCs, respectively. CFSE, carboxyfluorescein diacetate succinimidyl ester.

Understanding the action of these reagents, we next examined their effects on the proliferation of CD4^+ ^T cells. We found after stimulation with anti-CD3/CD28 that CD200-Fc did not cause a significant change in cellular proliferation (evaluated by the cell division index) in either SLE patients or HCs (*P *> 0.05; Figure 3A). However, anti-CD200R1 promoted anti-CD3/CD28-stimulated proliferation of SLE CD4^+ ^T cells in a concentration-dependent manner. In contrast, no effect was observed in HCs (Figure 3B). The cell division indexes in SLE T cells stimulated with anti-CD3/CD28 plus control IgG and with anti-CD3/CD28 plus anti-CD200R1 antibody at 20 ng/ml or 100 ng/ml were 0.87 ± 0.54, 1.43 ± 0.92, and 2.34 ± 1.85, respectively (*P *< 0.05). From this result, we concluded that antagonistic anti-CD200R1 antibody promoted T-cell receptor-driven proliferation of CD4^+ ^T cells in SLE patients, implying that endogenous CD200-CD200R1 interactions limited T-cell proliferation in SLE patients.

### CD200 reduces CD4^+ ^T-cell differentiation into T-helper type 17 cells

**Figure 4:**
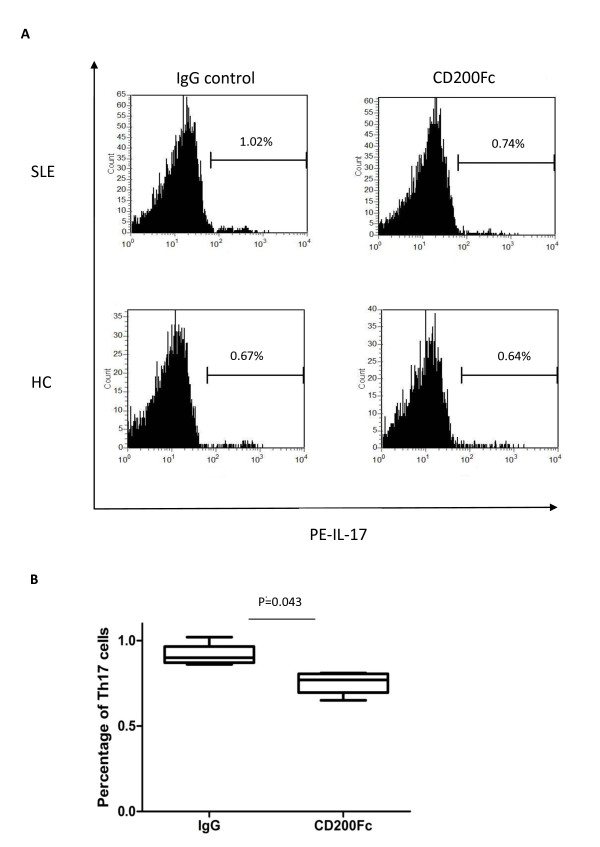
**CD200Fc reduced T-helper type 17 cell differentiation in systemic lupus erythematosus patients**. **(A) **Peripheral blood mononuclear cells from systemic lupus erythematosus (SLE) patients (*n *= 9) or healthy controls (HCs) (*n *= 6) were co-cultured with CD200Fc for 48 hours. PE, phycoerythrin. **(B) **Percentages of T-helper type 17 (Th17) cells detected by flow cytometry. Numbers indicate the percentage of Th17 cells in CD4^+ ^T cells.

The CD200/CD200R1 pathway has been suggested to affect the balance of cytokines, by repressing IL-2 (IFNγ) production and promoting IL-4 (IL-10) production, and to participate in transplantation tolerance [27]. We therefore investigated whether CD200-CD200R1 signaling could affect human CD4^+ ^T-cell differentiation. We found that, while the effect of the CD200-CD200R1 pathway on the T cells in HCs was negligible (see Additional file 6), CD200-Fc but not anti-CD200R1 reduced the percentage of Th17 cells (*P *< 0.05; Figure 4) in SLE patients, suggesting a role for CD200/CD200R signaling in regulating Th17 cell differentiation.

### CD200 signaling rescues the defective generation of CD4^+^CD25^high^FoxP3^+ ^T cells in SLE patients

**Figure 5:**
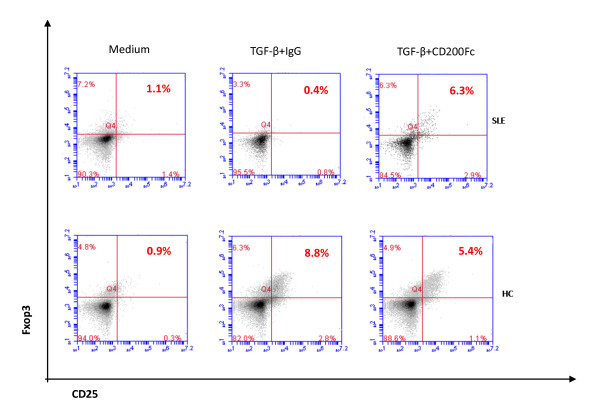
**Defective transforming growth factor beta-induced CD4^+^CD25^high^FoxP3^+ ^T cells were rescued by CD200Fc**. Defective transforming growth factor beta (TGF-β)-induced CD4^+^CD25^high^FoxP3^+ ^T cells were rescued from peripheral CD4^+^CD25^- ^T cells in systemic lupus erythematosus (SLE) patients by CD200Fc. Peripheral CD4^+^CD25^- ^T cells were sorted from peripheral blood mononuclear cells of SLE patients or healthy controls (HCs), cultured in complete RPMI 1640 medium combined with anti-CD3/CD28, IL-2 and TGF-β in the absence or presence of CD200Fc for 7 days. Data are representative of five experiments.

In a mouse collagen-induced arthritis model, the interaction between CD200 and CD200R1 resulted in direct suppression of autoreactivity, and fostered the development of Foxp3^+ ^regulatory T cells [28]. TGF-β induces *Foxp3 *gene expression in T-cell receptor-stimulated CD4^+^CD25^- ^naïve T cells, which mediates their transition toward a regulatory T-cell phenotype with potent immunosuppressive potential [29]. We therefore examined whether CD200 played a role in the induction of Tregs in SLE patients. CD4^+^CD25^- ^T cells were sorted from PBMC of SLE or HCs and were cultured with anti-CD3/CD28, IL-2 and TGF-β in the absence or presence of CD200-Fc or anti-CD200R1 for 7 days. We found that TGF-β induced CD4^+^CD25^high^FoxP3^+ ^T cells from peripheral CD4^+^CD25^- ^T cells in HCs, but not in SLE patients. Notably, CD200Fc but not anti-CD200R1 rescued the defective induction of CD4^+^CD25^high^FoxP3^+ ^T cells in SLE patients (Figure 5). In SLE patients, the percentages of induced CD4^+^CD25^high^Foxp3^+ ^T cells in TGF-β + IgG and TGF-β + CD200Fc groups were 0.93 ± 0.50% and 6.23 ± 0.72% (*P *= 0.013). We therefore concluded that the defective generation of CD4^+^CD25^high^FoxP3^+ ^T cells in SLE patients could be rescued by enhanced CD200 signaling. This result suggests that the decreased expression of CD200R1 in SLE may contribute to the defective generation of Tregs.

### Increased lymphocyte apoptosis with upregulation of CD200 expression in SLE

**Figure 6:**
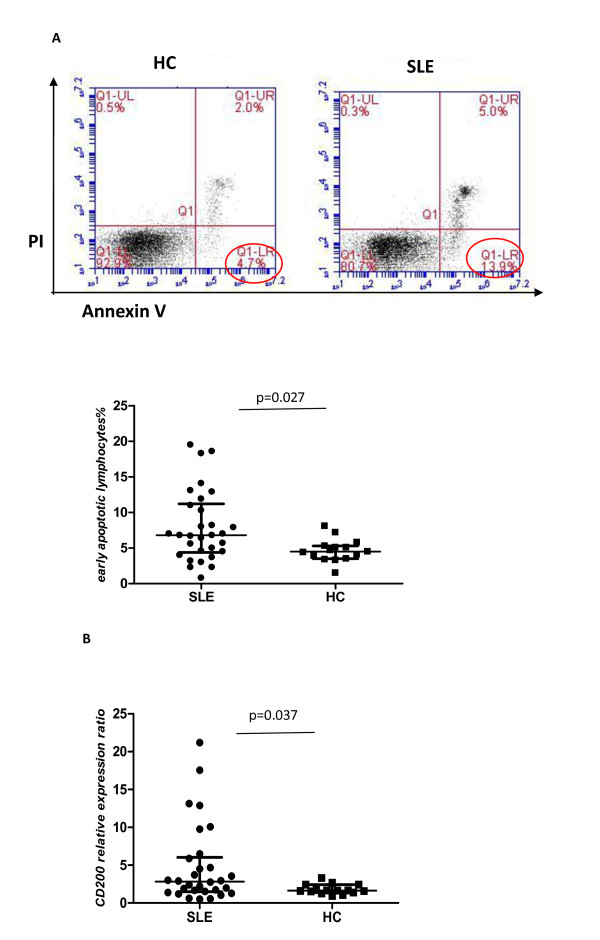
**Increased lymphocyte apoptosis with upregulation of CD200 expression in systemic lupus erythematosus**. **(A) **Percentage of spontaneous early apoptotic lymphocytes (annexin V^+^PI^-^) in peripheral blood mononuclear cells from systemic lupus erythematosus (SLE) patients (*n *= 30) was significantly higher than in healthy controls (HCs) (*n *= 15) (median 6.80, interquartile range 4.38 to 11.23 vs. 4.50, 3.50 to 5.30; *P *= 0.027). **(B) **CD200 relative expression ratio, defined as the percentage of CD200 positivity on early apoptotic cells to live cells, was significantly increased in SLE patients compared with HCs (median 2.83, interquartile range 1.50 to 6.04 vs. 1.64, 1.38 to 2.42; *P *= 0.037). PI, propidium iodide.

**Figure 7:**
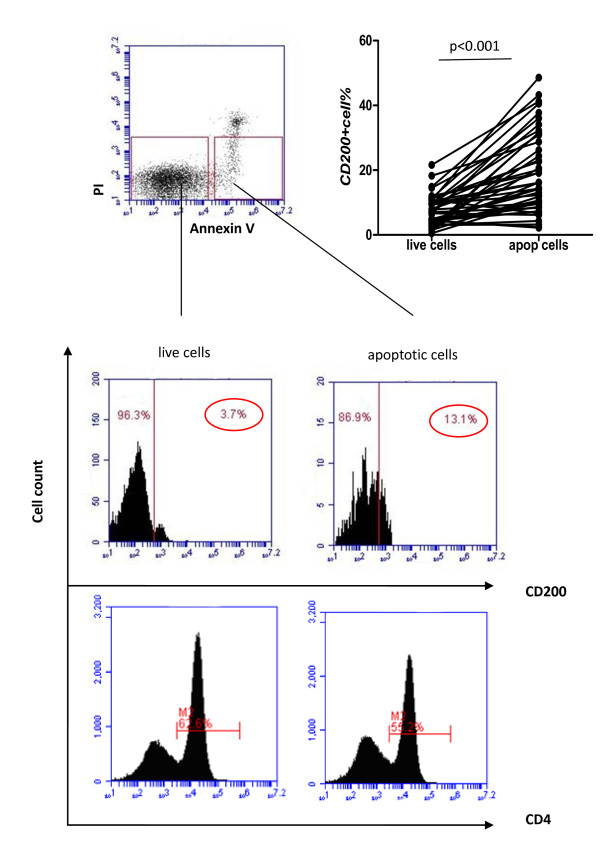
**CD200 expression on early apoptotic cells was higher than on live cells**. CD200 expression but not CD4 expression on early apoptotic cells was significantly higher than on live cells (annexin V^-^PI^-^) (*n *= 45) (median 14.5, interquartile range 8.5 to 28.7 vs. 6.30, 3.60 to 9.70; *P *< 0.001). PI, propidium iodide.

Next we were interested in determining whether CD200-CD200R interactions might also affect the activity of dendritic cells in SLE, and specifically their capacity to interact with apoptotic cells. Increased lymphocyte apoptosis and defective phagocytic removal of apoptotic cells have been suggested to contribute to the development of SLE [30-32]. We therefore initially examined whether CD200 expression by apoptotic cells was abnormal in SLE. Consistent with previous studies [4,5,33], we demonstrated that the percentage of spontaneous early apoptotic lymphocytes (denoted as annexin V^+^PI^-^) in PBMC from SLE patients was significantly higher than that in HCs (Figure 6A). Interestingly, we found that CD200 expression by early apoptotic cells was significantly higher compared with that expressed by live cells (annexin V^-^PI^-^), especially in SLE patients. By comparison, there was no increase in CD4 expression on apoptotic T cells detected with a mAb labeled with the same fluorochrome used to detect CD200 (Figure 7). The CD200 relative expression ratio, defined as the ratio of CD200 positivity on early apoptotic cells compared with live cells, was significantly increased in SLE patients compared with HCs (Figure 6B).

**Figure 8:**
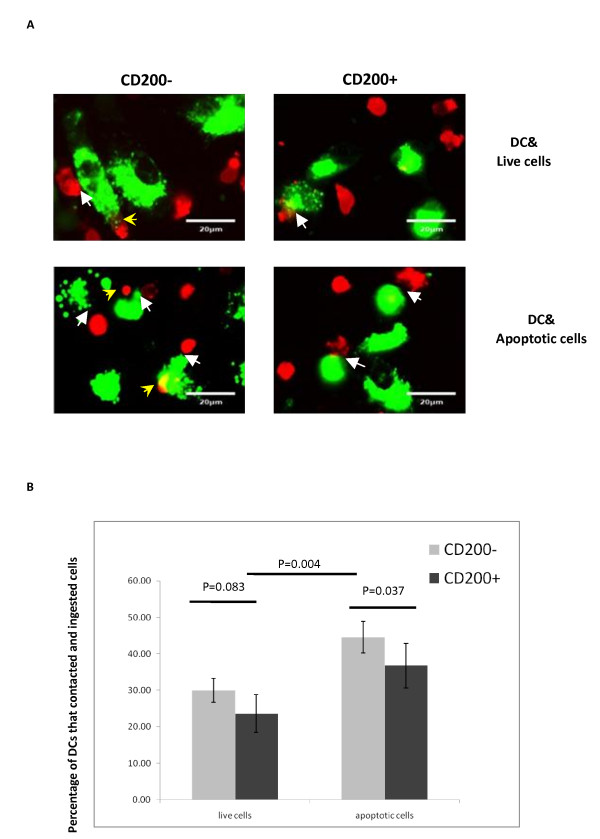
**CD200 expression on apoptotic cells affected their binding and phagocytosis by dendritic cells**. **(A) **Immature dendritic cells (DCs) (stained with PKH67, green) were incubated with target cells pre-labeled with PKH26 (red) and examined under immunofluorescence microscopy. White arrowheads, target cells attached to immature DCs; yellow arrowheads, target cells engulfed by immature DCs. Upper left, CD200^- ^live cells; upper right, CD200^+ ^live cells; lower left, CD200^- ^apoptotic cells; lower right, CD200^+ ^apoptotic cells. **(B) **Quantification of the percentages of DCs that bound and ingested target cells by immunofluorescence microscopy. The percentages were calculated by counting all the cells in 10 microscope fields (average 40 to 50 cells each microscope field and 400 to 500 cells were recorded in total).

**Figure 9:**
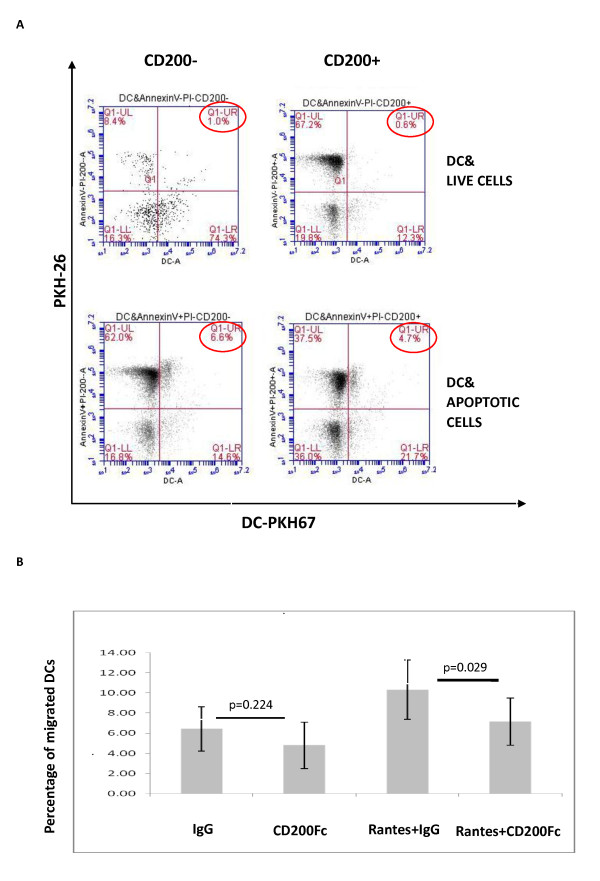
**CD200Fc reduced dendritic cell migration and ingestion of early apoptotic cells**. **(A) **Quantification of the percentages of dendritic cells (DCs) that ingested target cells by flow cytometry. PKH26/PKH67 double-positive cells indicated DCs that ingested target cells. **(B) **Percentage of DCs that migrated by transwell experiment (*n *= 5). PI, propidium iodide; RANTES, regulated on activation, normal T-cell expressed and **secreted (OK)**.

We next investigated whether CD200 expression on apoptotic cells affected their binding and phagocytosis by DCs. We obtained immature DCs by culturing human monocytes with IL-4 and GM-CSF for 6 days [34]. The immature DCs (PKH67-labeled) were then co-cultured for 3 hours with different target cell populations (PKH26-labeled), including CD200^+^/CD200^- ^apoptotic cells induced by irradiation and CD200^+^/CD200^- ^live cells, and were examined for cellular binding and uptake. We found that the proportion of DCs which bound and ingested apoptotic cells was higher compared with live cells. Importantly, CD200 expression on the target cells was associated with reduced binding and phagocytosis of apoptotic cells by DCs (Figures 8 and 9A). The percentages of DCs that bound and ingested CD200^- ^versus CD200^+ ^apoptotic cells were 44.54 ± 4.33% versus 36.76 ± 6.09% by fluorescence microscopy (*P *= 0.037). By flow cytometry, the percentages of DCs that ingested CD200^- ^versus CD200^+ ^apoptotic cells - demonstrated as PKH26 and PKH67 double-positive events - were 31.60 ± 22.98% versus 21.71 ± 20.20% (*P *= 0.046). The results suggested that CD200 expression on early apoptotic cells is associated with decreased binding and phagocytosis by DCs.

### CD200Fc inhibits dendritic cell migration

We were interested in whether CD200-CD200R interactions might affect other functional activities of DCs, and therefore examined the impact on DC migration. In initial experiments, we noted a potential effect of soluble CD200Fc itself in reducing spontaneous DC migration - although the effect was not statistically significant (IgG vs. CD200Fc, 6.21 ± 2.23% vs. 4.38 ± 2.42% migrated; *P *= 0.224). However, CD200Fc significantly blocked RANTES-induced DC migration (IgG vs. CD200Fc, 10.32 ± 2.94% vs. 7.16 ± 2.36% migrated; *P *= 0.029; Figure 9B).

## Discussion

Despite the data in animal models - including collagen-induced arthritis and experimental allergic encephalomyelitis - suggesting that CD200/CD200R1 may play a role in prevention of autoimmune diseases, information on the role of the CD200/CD200R axis in human diseases - especially in SLE - is very limited. Our study demonstrated that the percentage of CD200^+ ^cells in CD4^+ ^T cells, plasmacytoid DCs and myeloid DCs of SLE patients was significantly higher than that for HCs. In addition, serum levels of CD200 in SLE patients were also significantly higher than those for HCs.

As CD200 lacks an intracellular signaling motif, most - if not all - of its immunological function relates to its capacity to engage and signal via its receptors, of which CD200R1 seems to be most prominent. Functional studies confirmed this by showing that CD200Fc induced phosphorylation of DOK2 in CD4^+ ^T cells. Notably, we found that CD200R1 expression in SLE patients was significantly lower than HCs in CD4^+ ^T cells and DCs. The dysregulated expression of CD200/CD200R1 in SLE had functional consequences since CD4^+ ^T-cell proliferation was increased by blocking CD200R1 with specific antibody, whereas DC migration and Th17 cell differentiation were decreased and Treg generation was enhanced by engaging CD200R with CD200Fc. These results are all consistent with the conclusion that the deranged expression of both CD200 and CD200R1 in SLE contributes to the functional abnormalities characteristic of this autoimmune disease. Notably, most of the activity of CD200-CD200R1 engagement is usually believed to relate to inhibiting the activity of myeloid cell function [35]. However, we found that CD200R1 expression was also decreased on CD4^+ ^T cells and at least the activity in regulating Tregs appeared to involve a direct effect on T cells. These findings suggest a broader spectrum of activity of CD200R1 signaling than has previously been appreciated.

Overproduction of autoantibodies in SLE is believed to be caused by insufficient removal of apoptotic cells and material by macrophages and DCs. Our study demonstrated that SLE patients had a higher proportion of spontaneous early apoptotic lymphocytes compared with HCs. The amount of apoptotic material in SLE patients may exceed the capacity of macrophages to remove it, allowing DCs to become involved in the process of apoptotic cell clearance. Under these circumstances, DCs can become either tolerogenic or stimulatory, depending on the nature of the receptors employed and the available cytokines. As CD200 expression on early apoptotic lymphocytes was increased in SLE patients, we examined whether the increased expression of CD200 on early apoptotic lymphocytes might have had an effect on their binding and uptake by DCs. We demonstrated that early apoptotic cells were more likely to be bound and engulfed by DCs than living cells. The explanation for this could be that although early apoptotic cells remain morphologically intact, specific signals - such as expression of lysophosphatidylcholine - were upregulated on the cell surface, which mediated recognition by DCs and macrophages [36,37]. Our study also revealed that the binding and phagocytosis of early apoptotic cells that were CD200-positive were lower than those that did not express CD200, suggesting that CD200 expression in SLE could provide a signal to DCs - presumably by binding CD200R, which limits their capacity to bind and ingest apoptotic material. Aberrant expression of CD200 may therefore contribute to the decreased clearance of apoptotic material found in SLE.

To function, CD200 needs to bind to CD200R on cell surfaces. Our data confirmed that T cells expressed CD200R1. Since a previous animal study suggested that CD200/CD200R signaling had an effect on cytokine balance [27], we investigated whether CD200/CD200R1 could affect the balance of T-cell subsets in SLE. We found that CD200Fc reduced Th17 cell differentiation in SLE, but not in HCs. These results suggest that Th17 cell differentiation in SLE may be regulated by engagement of CD200R, such that signaling through this pathway limited Th17 cell differentiation. It is possible that the downregulation of CD200R in SLE resulted in less regulation of Th17 cell differentiation, which could be corrected by the increased availability of CD200. On the contrary, anti-CD3/CD28-stimulated T-cell proliferation was promoted by antagonistic anti-CD200R1 in a concentration-dependent manner in SLE patients but not HCs, suggesting that anti-CD200R1 may block the endogenous signal provided by increased expression of CD200 and, thereby, permit increased CD4^+ ^T-cell proliferation. In summary, these results indicate that the CD200-CD200R1 pathway exerts a number of regulatory influences on T-cell function, either directly or through the action of DCs, and that the dysregulation of surface expression of these molecules may contribute to some of the immunoregulatory abnormalities characteristic of SLE.

In untreated active SLE patients, the proportion of CD4^+^CD25^high^FoxP3^+ ^Tregs was significantly lower than in HCs (see Additional file 7). Pallasch and colleagues demonstrated that antagonistic anti-CD200 antibody could promote chronic lymphocytic leukemia cell-induced proliferation of antigen-specific T cells and reduce the proportion of CD4^+^CD25^high^FoxP3^+ ^cells [38]. Gorczynski and colleagues showed that, in BL/6 bone-marrow cells, anti-CD200R2/3 mAb (not CD200R1) could promote the development of tolerogenic DCs through a TGF-β-dependent (but not IL-10) and CTLA-4-dependent pathway, induce more CD4^+^CD25^high^FoxP3^+ ^Tregs, and inhibit the mixed lymphocyte reaction in a MHC-restricted and antigen-specific manner [39]. These results all suggested that the activation of the CD200/CD200R axis could exert an immunosuppressive function via promoting Treg proliferation and inhibiting effector T-cell function. Our study found that TGF-β induced generation of CD4^+^CD25^high^FoxP3^+ ^T cells in HCs, whereas this was not seen in SLE patients. This finding is consistent with a previous study that demonstrated defective expression of TGF-β signal transduction molecules in most SLE patients [40]. Interestingly, we found that the addition of CD200Fc rescued the defective generation of CD4^+^CD25^high^FoxP3^+ ^T cells in SLE patients, indicating that CD200 could intervene in the TGF-β signaling pathway and promote Treg generation. This effect appeared to be directly mediated by T cell-T cell interaction since these studies were carried out with sorted T cells. Specific signals and cytokines mediate the differentiation of Tregs and Th17 cells [41,42]. The current data imply that signaling through CD200R1 may be one important influence on these pathways of T-cell differentiation. Increased signaling through CD200R1 may bias toward Tregs and away from Th17 cells, and thus may be beneficial in SLE.

Downregulation of CD200R1 in SLE may contribute to impaired generation of regulatory signals, and increased production of CD200 *in vivo *could bind to other receptors such as CD200R2 to CDR200R4 [14], thereby transmitting stimulatory signals leading to the enhanced differentiation of Th17 cells, as has been reported [43]. Moreover, it has been reported that CD200 engagement of CD200R1 could induce tolerogenic DCs, which in turn could promote differentiation of Tregs [39,44,45].

In our study, however, experiments were carried out with purified T cells, making this a less probable explanation for the findings. CD200R1 expression by DCs was also downregulated in SLE, however, suggesting that reduced generation of tolerogenic DCs in the context of decreased Tregs could contribute to unregulated development of Th17 cells.

## Conclusions

Taking the results together, we have demonstrated in SLE patients that the number of CD200^+ ^cells as well as the serum level of CD200 were significantly higher than in HCs, whereas CD200R1 expression was significantly lower than in HCs, especially in CD4^+ ^T cells and DCs. In addition, in SLE patients, exogenous CD200Fc reduced the proportion of Th17 cells and rescued the defective generation of CD4^+^CD25^high^FoxP3^+ ^T cells, whereas anti-CD200R1 antibody promoted anti-CD3/CD28-induced CD4^+ ^T-cell proliferation. Moreover, we found that CD200 on early apoptotic cells was increased in SLE patients and may serve to limit their binding and phagocytosis by DCs. These data collectively indicate that CD200 and CD200R1 expression and function are abnormal in SLE and may contribute to the immunologic abnormalities in this autoimmune disease.

## Abbreviations

CD200R1: CD200 receptor 1; DC: dendritic cell; DOK2: downstream of tyrosine kinase 2; dsDNA: double-stranded DNA; ELISA: enzyme-linked immunosorbent assay; FCS: fetal calf serum; GM-CSF: granulocyte-macrophage colony-stimulating factor; HC: healthy control; IFN: interferon; IL: interleukin; mAb: monoclonal antibody; PBMC: peripheral blood mononuclear cells; PBS: phosphate-buffered saline; PI: propidium iodide; RANTES: regulated on activation, normal T-cell expressed and secreted; SLE: systemic lupus erythematosus; TGF-β: transforming growth factor beta; Th17: T-helper type 17; TNF: tumor necrosis factor; Treg: regulatory T cell.

## Competing interests

The authors declare that they have no competing interests.

## Authors' contributions

YL, L-dZ, and L-sT developed the study, analyzed the data and drafted the manuscript. S-nQ, YR, LZ, XD, YC, and Y-xW participated in the data collection and performed the data analysis. WZ, X-fZ, F-cZ, and F-lT participated in the enrollment of patients. XZ and PEL designed the study, participated in the data analysis and manuscript preparation. D-nB, WH, and X-tC participated in coordination of the study. All authors read and approved the final manuscript.

## Supplementary Material

Additional file 1 **Supplementary Table S1 presenting characteristics of the SLE patients (*n *= 161)**. Click here for file

Additional file 2 **Supplementary Figure S1 presenting fluorescence-activated cell sorting (FACS) plots specifically showing CD200 expression in CD11c^-^CD123^high ^plasmacytoid DCs (pDC) (Gate 2) and CD11c^+^CD123^- ^myeloid DCs (mDC) (Gate 3)**. Click here for file

Additional file 3 **Supplementary Figure S2 showing the serum CD200 level did not correlate with the Systemic Lupus Erythematosus Disease Activity Index score, anti-dsDNA, IFNα, IL-6, or B-cell activating factor belonging to the TNF family (BAFF) in SLE patients**. Click here for file

Additional file 4 **Supplementary Figure S3 showing expression of CD200R1 in naïve T cells (CD4^+^CD45RA^+^) and memory T cells (CD4^+^CD45RO^+^) of SLE patients tended to decrease compared with HCs, although it did not reach statistical significance (*P *= 0.50 and 0.11, respectively)**. Naïve T cells had less CD200R1 expression than memory T cells both in HCs and SLE patients (*P *= 0.0003 and *P *< 0.001). Click here for file

Additional file 5 **Supplementary Figure S4 showing immunoblot analysis of the expression of DOK2 (left) and p-DOK2 (right) in CD4^+ ^T cells**. CD200Fc induced phosphorylation of DOK2 (lane 2 on right). Click here for file

Additional file 6 **Supplementary Table S2 showing the effect of CD200 signaling on the differentiation of T-helper cell subsets**. Click here for file

Additional file 7 **Supplementary Figure S5 showing the proportion of CD4^+^CD25^high^Foxp3^+ ^T cells in new-onset active untreated SLE patients was significantly lower than in HCs (median 1.42, interquartile range 0.75 to 2.43 vs. 2.79, 1.95 to 4.52; *P *= 0.014)**. All cells plotted were CD4^+^. Click here for file
