# Supplementary material for: Aberrant CD200/CD200R1 expression and function in systemic lupus erythematosus contributes to abnormal T-cell responsiveness and dendritic cell activity
Source: Arthritis Res Ther. 2012 May 23;14(3):R123. doi: 10.1186/ar3853 (PMC3446504; doi:10.1186/ar3853)
Supplement: Additional file 1 — Supplementary Table S1 presenting characteristics of the SLE patients (n = 161). [file ar3853-S1.DOC]

**Table s1**. The characteristics of the SLE patients(n=161)

| Female/male(n) |  | 161/0 |
| --- | --- | --- |
| Age,mean±SD (years) |  | 29.0±10.2(12-55) |
| Disease duration, mean±SD (days) |  | 23.0±33.0(10-180) |
| SLEDAI, mean±SD |  | 12.2±6.3(1-29) |
| Lupus nephritis(proteinuria≥0.5g/24h)(n,%) |  | 69 ( 42.9%) |
| Neuropsychiatric manifestations(n,%) |  | 19 (11.8%) |
