# Supplementary material for: Aberrant CD200/CD200R1 expression and function in systemic lupus erythematosus contributes to abnormal T-cell responsiveness and dendritic cell activity
Source: Arthritis Res Ther. 2012 May 23;14(3):R123. doi: 10.1186/ar3853 (PMC3446504; doi:10.1186/ar3853)
Supplement: Additional file 2 — Supplementary Figure S1 presenting fluorescence-activated cell sorting (FACS) plots specifically showing CD200 expression in CD11c-CD123high plasmacytoid DCs (pDC) (Gate 2) and CD11c+CD123- myeloid DCs (mDC) (Gate 3). [file ar3853-S2.DOC]

Figure s1

CD11c

CD123

CD123

CD200


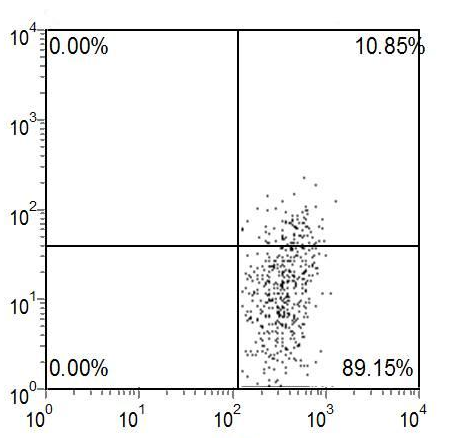

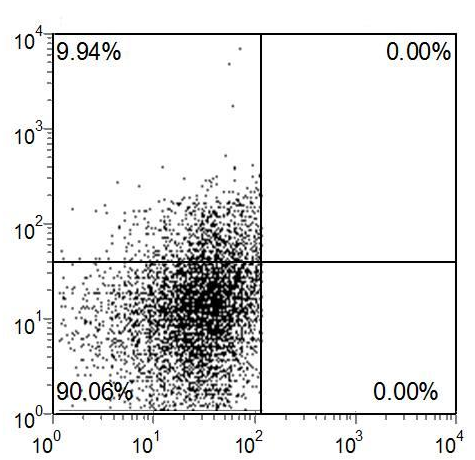

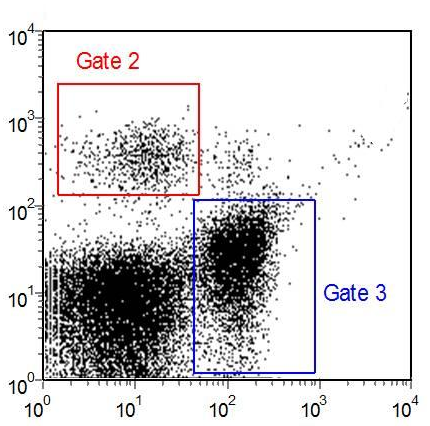


CD11c^-^CD123^high^ pDC

CD11c^+^CD123^-^ mDC

Figure s1 FACS plots specifically showing CD200 expression in CD11c^-^CD123^high^ plasmacytoid DC (pDC) (Gate 2) and CD11c^+^CD123^-^ myeloid DC (mDC) (Gate 3).
