# Supplementary material for: Aberrant CD200/CD200R1 expression and function in systemic lupus erythematosus contributes to abnormal T-cell responsiveness and dendritic cell activity
Source: Arthritis Res Ther. 2012 May 23;14(3):R123. doi: 10.1186/ar3853 (PMC3446504; doi:10.1186/ar3853)
Supplement: Additional file 3 — Supplementary Figure S2 showing the serum CD200 level did not correlate with the Systemic Lupus Erythematosus Disease Activity Index score, anti-dsDNA, IFNα, IL-6, or B-cell activating factor belonging to the TNF family (BAFF) in SLE patients. [file ar3853-S3.DOC]

Figure s2


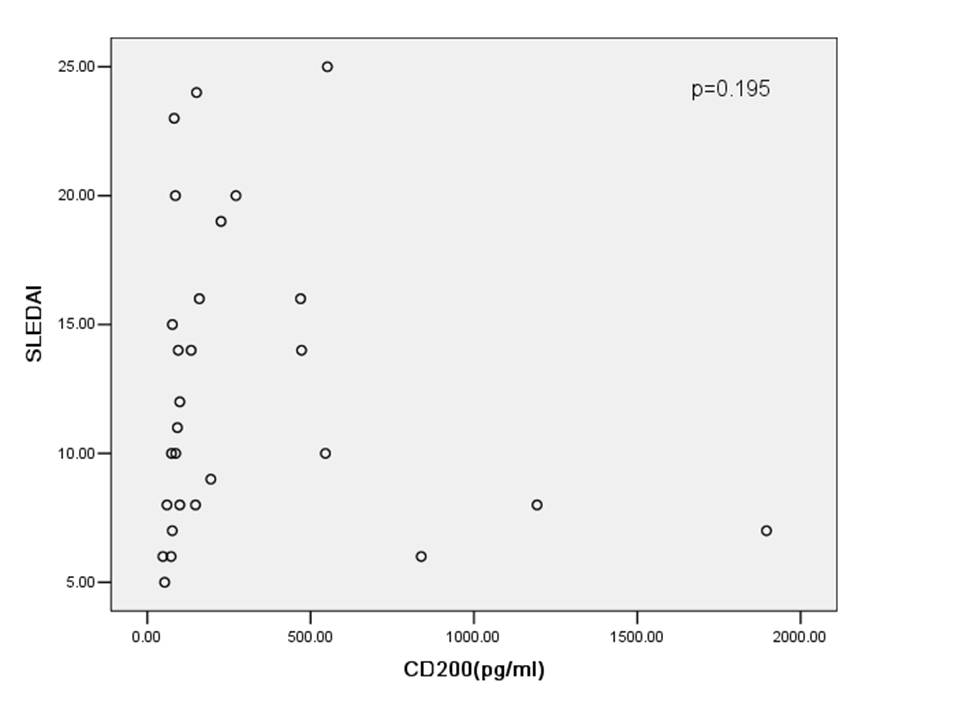

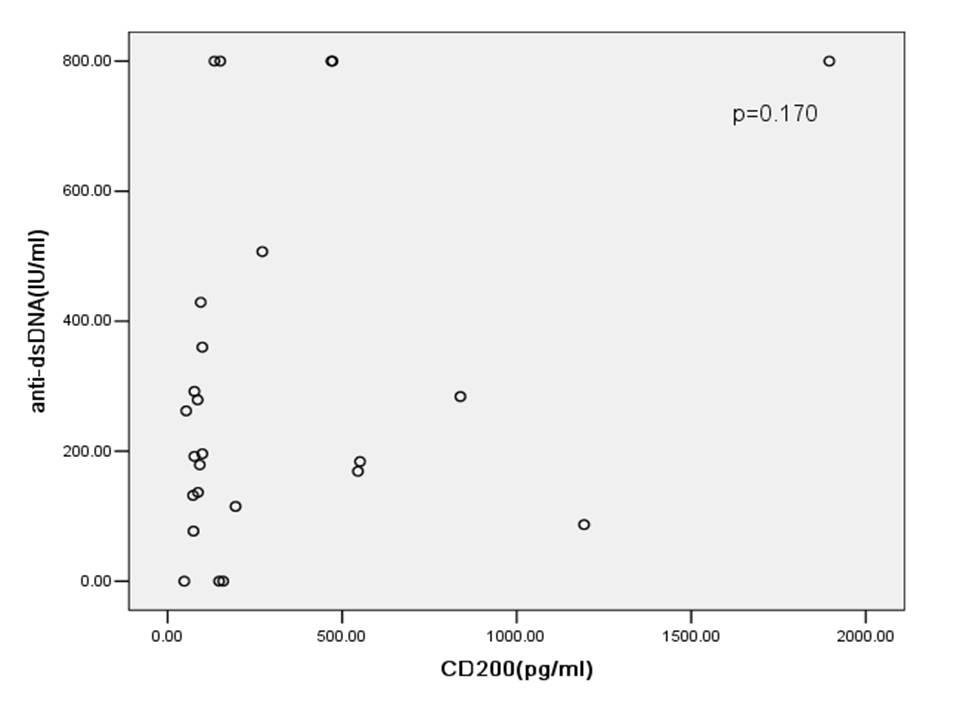

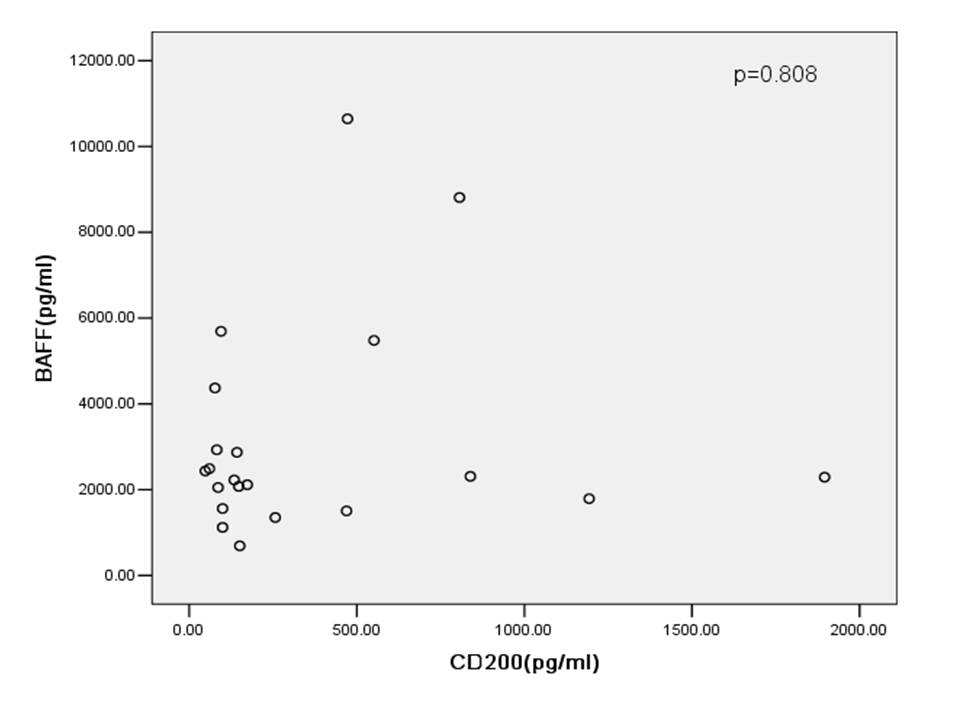

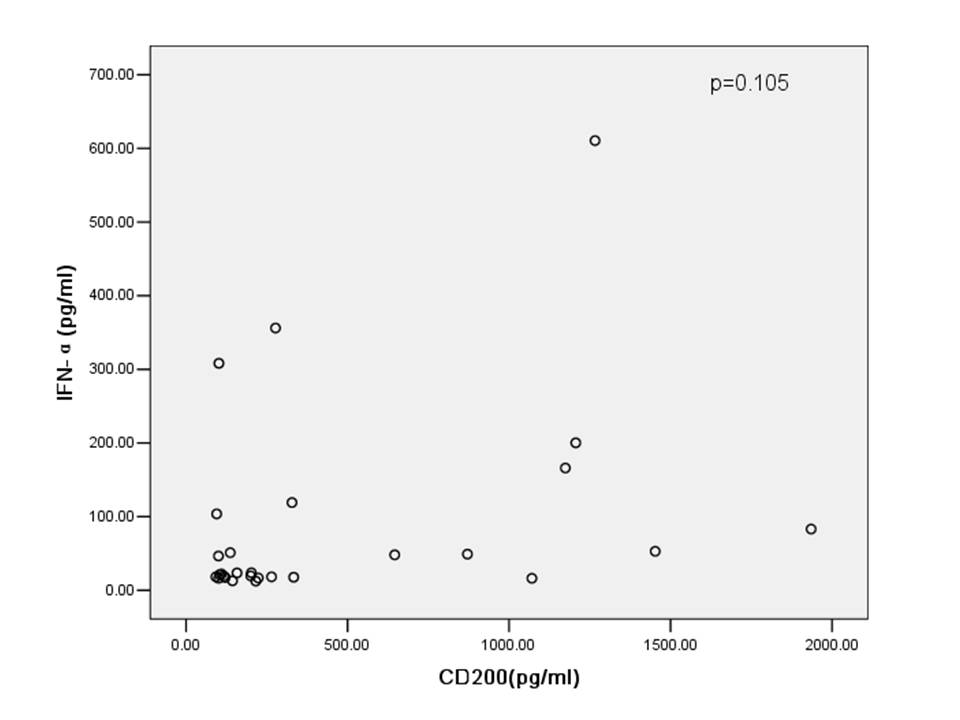

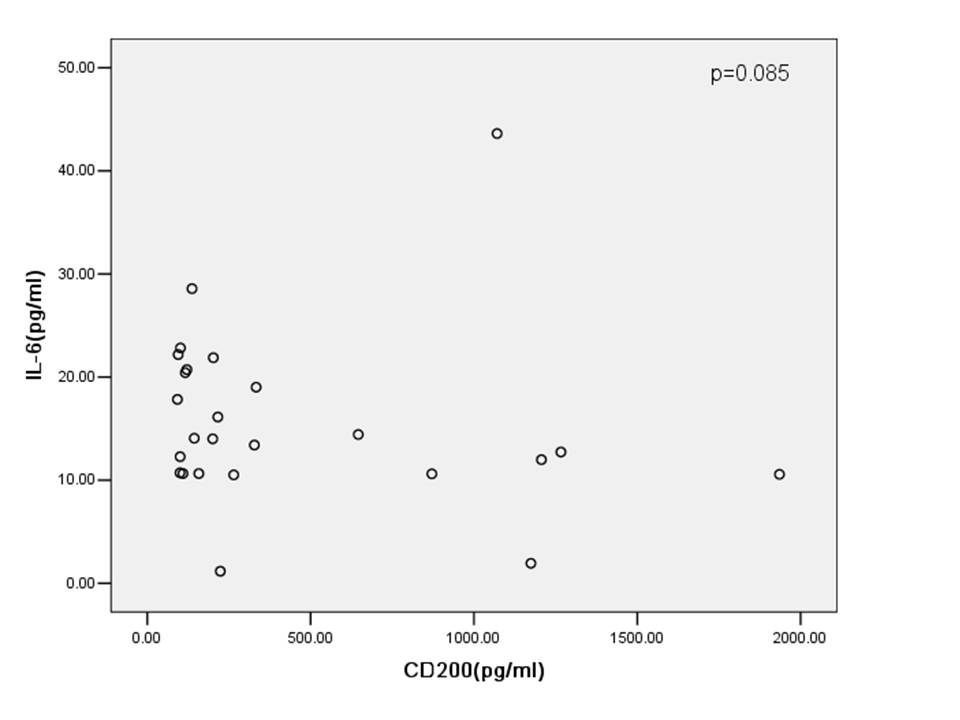


**Figure s2**

Serum CD200 level did not correlate with SLEDAI score，anti-dsDNA , IFN-α，IL-6, or BAFF(B-cell activating factor belonging to tumour necrosis factor family) in SLE patients.
