# Supplementary material for: Aberrant CD200/CD200R1 expression and function in systemic lupus erythematosus contributes to abnormal T-cell responsiveness and dendritic cell activity
Source: Arthritis Res Ther. 2012 May 23;14(3):R123. doi: 10.1186/ar3853 (PMC3446504; doi:10.1186/ar3853)
Supplement: Additional file 4 — Supplementary Figure S3 showing expression of CD200R1 in naïve T cells (CD4+CD45RA+) and memory T cells (CD4+CD45RO+) of SLE patients tended to decrease compared with HCs, although it did not reach statistical significance (P = 0.50 and 0.11, respectively). Naïve T cells had less CD200R1 expression than memory T cells both in HCs and SLE patients (P = 0.0003 and P < 0.001). [file ar3853-S4.DOC]

Figure s3

**Figure s3** Expression of CD200R1 in naïve T cells（CD4^+^CD45RA^+^）and memory T cells (CD4^+^CD45RO+) of SLE patients tended to decrease comparing with healthy controls (HC) albeit it did not reach statistical significance (p=0.50 and 0.11，respectively). Naïve T cells had less CD200R1 expression than memory T cells both in HC and SLE patients (p=0.0003 and p<0.001).
