# Supplementary material for: Aberrant CD200/CD200R1 expression and function in systemic lupus erythematosus contributes to abnormal T-cell responsiveness and dendritic cell activity
Source: Arthritis Res Ther. 2012 May 23;14(3):R123. doi: 10.1186/ar3853 (PMC3446504; doi:10.1186/ar3853)
Supplement: Additional file 5 — Supplementary Figure S4 showing immunoblot analysis of the expression of DOK2 (left) and p-DOK2 (right) in CD4+ T cells. CD200Fc induced phosphorylation of DOK2 (lane 2 on right). [file ar3853-S5.DOC]

Figure s4

DOK2

IgGFc

CD200Fc


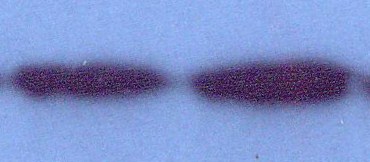


p-DOK2


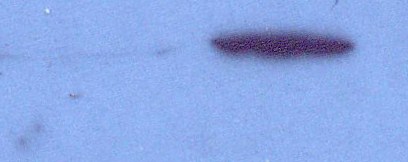


CD200Fc

IgGFc

**Figure s4** Immunoblot analysis of the expression of DOK2(left) and p-DOK2(right) in CD4^+^ T cells. CD200Fc induced phosphorylation of DOK2(lane 2 in the right).
