# Supplementary material for: Aberrant CD200/CD200R1 expression and function in systemic lupus erythematosus contributes to abnormal T-cell responsiveness and dendritic cell activity
Source: Arthritis Res Ther. 2012 May 23;14(3):R123. doi: 10.1186/ar3853 (PMC3446504; doi:10.1186/ar3853)
Supplement: Additional file 6 — Supplementary Table S2 showing the effect of CD200 signaling on the differentiation of T-helper cell subsets. [file ar3853-S6.DOC]

**Table s2**. Effect of CD200 signaling on the differentiation of Th subsets.

|  | HC(n=6) | | | | SLE(n=9) | | |  |
| --- | --- | --- | --- | --- | --- | --- | --- | --- |
|  | Isotype | CD200-Fc | Anti-CD200R1 | isotype | | CD200-Fc | Anti-CD200R1 | |
| Th1(CD4^+^IFN-γ^+^） | 11.01,7.90-16.56 | 11.85,8.24-17.37 | 9.15,6.45-11.99 | 4.98,4.22-8.05 | | 5.57,4.22-9.9 | 5.29,4.55-5.31 | |
| Th2  (CD4^+^  IL-4^+^) | 0.42,0.28-0.54 | 0.43,0.32-0.55 | 0.33-0.31-0.60 | 0.60,0.43-1.68 | | 0.58,0.42-2.69 | 0.42,0.27-0.82 | |
| Th17  (CD4^+^  IL-17^+^) | 0.84,0.68-1.14 | 0.89,0.64-1.13 | 0.90,0.70-1.70 | 0.90,0.87-0.97 | | 0.77,0.70-0.81* | 0.92,0.83-1.26 | |

Data are shown as median，interquartile range. *p<0.05.
