# Supplementary material for: Aberrant CD200/CD200R1 expression and function in systemic lupus erythematosus contributes to abnormal T-cell responsiveness and dendritic cell activity
Source: Arthritis Res Ther. 2012 May 23;14(3):R123. doi: 10.1186/ar3853 (PMC3446504; doi:10.1186/ar3853)
Supplement: Additional file 7 — Supplementary Figure S5 showing the proportion of CD4+CD25highFoxp3+ T cells in new-onset active untreated SLE patients was significantly lower than in HCs (median 1.42, interquartile range 0.75 to 2.43 vs. 2.79, 1.95 to 4.52; P = 0.014). All cells plotted were CD4+. [file ar3853-S7.DOC]

（A）

HC

SLE

Figure s5

(B)


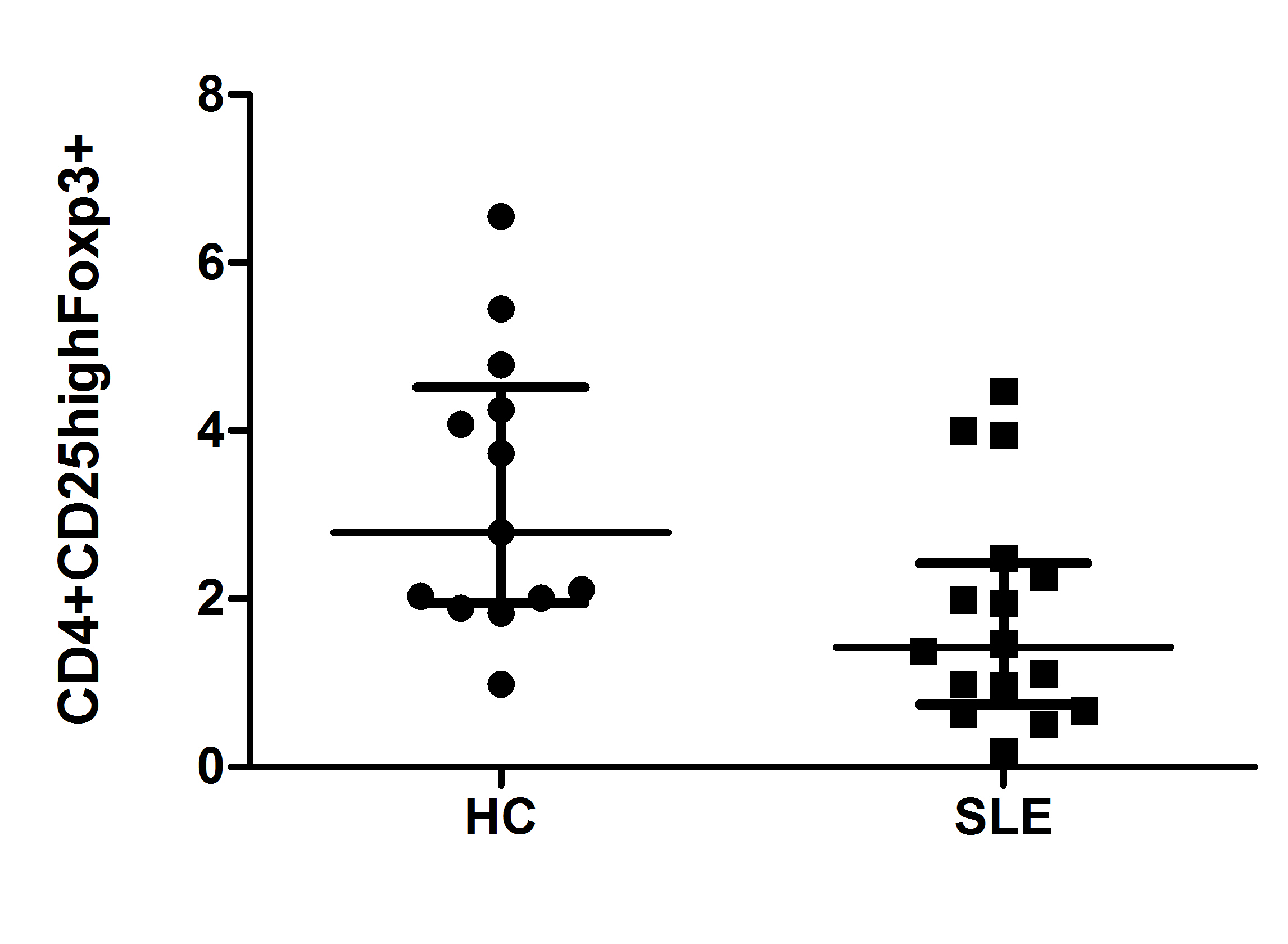

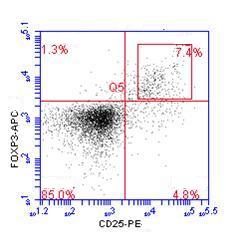

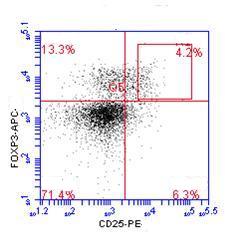


**Figure s5** The proportion of CD4^+^CD25^high^Foxp3^+^T cells in new onset active untreated SLE patients was significantly lower than health controls (HCs) (median 1.42, interquartile range 0.75-2.43 vs. 2.79, 1.95-4.52)(p=0.014). All cells plotted were CD4^+^.
